# Stratified medicine in practice: review of predictive biomarkers in European Medicines Agency (EMA) indications

**DOI:** 10.1186/1745-6215-12-S1-A16

**Published:** 2011-12-13

**Authors:** Kinga Malottki, Mousumi Biswas, Jon Deeks, Richard Riley, Charles Craddock, Lucinda Billingham

**Affiliations:** 1 MRC Midland Hub for Trials Methodology Research, University of Birmingham, Birmingham, UK; 2 Cancer Research UK Clinical Trials Unit, University of Birmingham, Birmingham, UK; 3 Public Health, Epidemiology and Biostatistics, University of Birmingham, Birmingham, UK; 4 Centre for Clinical Haematology, Queen Elizabeth Hospital, Birmingham, UK

## Objectives

Stratified medicine has been defined as using a biomarker to match a patient to a cohort that has exhibited a differential response to a treatment. This is important where the proportion of patients benefiting from treatment is low and possible adverse events can be serious. To maximise patient benefit, valid predictive biomarkers need to be used. Several trial designs have been proposed to evaluate the use of predictive biomarkers in clinical practice including: enrichment, stratified and biomarker-based strategy design.

Our aim was to review the EMA indications that include a predictive biomarker in order to investigate the type and strength of evidence considered sufficient for such decisions.

## Methods

We have undertaken a review to identify predictive biomarkers included in EMA indications, together with the supporting study designs and strength of evidence. The authorised, refused, withdrawn and pending decisions on the EMA website were reviewed in October 2010. Where predictive biomarkers were identified, data was collected on the details of the therapeutic indication and supporting evidence.

## Results

Fifteen predictive biomarkers were included in the indications of 18 drugs. For one biomarker the license was refused and for one withdrawn. Only three biomarkers were included in an indication before 2004. Five of the 18 drugs had an orphan designation.

Thirteen biomarkers for 10 drugs were included in indications for treatment of various cancers (including a range of haematological diseases, breast, colorectal, gastric and lung cancer). Two biomarkers were included in the indications of four drugs for the treatment of HIV infections.

The majority of identified studies were enrichment design or used a subgroup analysis (sometimes post hoc) to evaluate the predictive biomarker. One stratified and one marker- based strategy design study was identified.

## Conclusions

The specialties where predictive biomarkers were identified were limited to cancer and HIV infection. No predictive biomarkers have been identified in other specialties where treatments are often effective only in a relatively small subgroup of patients (such as mental health).

Our review found that evidence from subgroup analyses and studies with an enrichment design has been often considered sufficient to grant marketing authorisation. Such results would ordinarily be interpreted with caution due to underlying methodological limitations.

